# A multicentre, prospective, randomised, blinded clinical trial to compare some perioperative effects of buprenorphine or butorphanol premedication before equine elective general anaesthesia and surgery

**DOI:** 10.1111/evj.12442

**Published:** 2015-06-10

**Authors:** P. M. Taylor, H. R. Hoare, A. de Vries, E. J. Love, K. M. Coumbe, K. L. White, J. C. Murrell

**Affiliations:** ^1^ Taylor MonroeLittle DownhamCambridgeshireUK; ^2^ School of Veterinary Medicine and ScienceNottingham UniversitySutton BoningtonLeicestershireUK; ^3^ Animal Health TrustNewmarketSuffolkUK; ^4^ Equine First Opinion and Referral ClinicUniversity of BristolLangfordBristolUK; ^5^ Bell Equine ClinicMereworthKentUK; ^6^ School of Veterinary ScienceUniversity of BristolLangfordBristolUK

**Keywords:** horse, general anaesthesia, buprenorphine, butorphanol, opioid

## Abstract

**Reasons for performing study:**

Buprenorphine, a **μ**‐agonist opioid, has recently been licensed for equine use, but butorphanol, a **κ**‐agonist opioid, is more commonly used in horses. The effect of the 2 opioids has not previously been compared in a large clinical study.

**Objectives:**

To compare post operative analgesia and physiological variables in horses undergoing elective surgery following premedication with either buprenorphine or butorphanol in a conventional clinical setting.

**Study design:**

Multicentre, prospective, randomised, blinded clinical investigation.

**Methods:**

Eighty‐nine healthy horses admitted for elective surgery to one of 6 UK equine veterinary clinics were premedicated with acepromazine, a nonsteroidal anti‐inflammatory drug, and romifidine followed by intravenous (i.v.) buprenorphine or butorphanol. Anaesthesia was induced with diazepam/ketamine and maintained with isoflurane in oxygen. A range of surgical procedures were performed and supplementary anaesthetic agents given as required. Physiological variables were monitored during anaesthesia and pain, ataxia, sedation and vital function were assessed post operatively. Data were analysed using *t*‐tests, ANOVA, Mann–Whitney *U*‐test and Chi‐squared test as appropriate and P<0.05 was regarded as significant, except for multiple comparisons, when P<0.01 was used.

**Results:**

Surgery was carried out successfully in all cases and no mortality or serious morbidity occurred. Physiological variables remained within normal limits and all horses recovered successfully, most standing within 1 h of ceasing anaesthesia. There were no significant differences between groups in any variable except post operative pain when scores (simple descriptive scale) between 3 and 6 h were significantly lower after buprenorphine than after butorphanol.

**Conclusions:**

Horses experienced less post operative pain after buprenorphine than after butorphanol premedication. Compared with butorphanol, buprenorphine did not cause any different effects on vital function.

## Introduction

It is now widely accepted that horses should receive perioperative analgesia in the same way as other species 1. Opioids are often used to provide perioperative analgesia in small animals and man, as they are not only effective analgesics but also contribute to balanced anaesthesia by providing intrinsic analgesia during surgery. Opioids have been used to enhance sedation for many years 2, 3 but there is still some reluctance for their perioperative use in horses, leading to over‐reliance on nonsteroidal anti‐inflammatory drugs (NSAIDs) 4, 5. Opioids are often withheld in horses because of fear of locomotor stimulation and depression of intestinal motility 4, 6. Much evidence comes from studies of high doses in healthy research horses; opioids are less likely to cause side effects in animals experiencing clinical pain 7. Several studies investigating perioperative opioid use in horses have not shown an increased incidence of adverse events 8, 9, 10, suggesting that opioids could be more widely used for perioperative analgesia in horses.

It is generally accepted that **μ** opioid receptor agonists provide the best analgesia. However, butorphanol, a **κ** agonist, is still most commonly used in horses to provide analgesia and enhance sedation, although it is often regarded as a relatively ineffective analgesic. Butorphanol did not produce better post castration pain control than placebo 11, while in contrast buprenorphine, a partial **μ** agonist, was superior to both butorphanol 12 and placebo 13. Although morphine and methadone, pure **μ** agonists, would be expected to provide the best opiate analgesia and are quite commonly used in horses, they do not have appropriate UK market authorisation and there are no published clinical studies investigating the analgesic effect of systemic methadone or morphine in horses undergoing general anaesthesia for surgery. Buprenorphine has recently received UK market authorisation for analgesia and enhancement of sedation in horses, and its potential contribution to clinical equine anaesthesia requires evaluation. The hypothesis of this investigation was that buprenorphine would provide better post operative analgesia than butorphanol. The aim of the investigation was to compare the degree of post operative pain experienced by horses after elective surgery following premedication with either buprenorphine or butorphanol in a conventional clinical setting. A subsidiary aim was to evaluate any differences in physiological variables and other drug requirements.

## Materials and methods

The study was originally approved under Animal Test Certificate No. 14094/0006 and informed consent was obtained from the owner of each horse. Market authorisation for buprenorphine as Vetergesic Multidose^1^ was granted and ATC requirements relaxed during the course of investigation. Thereafter, restriction on the inclusion of common supplementary drugs such as NSAIDs and local anaesthetics was lifted.

### Horses

Horses admitted for elective surgery under general anaesthesia to one of 6 UK equine veterinary clinics were recruited. Horses over 6 months of age graded ASA 1 or 2 under the American Society of Anesthesiologists physical status classification system were eligible for inclusion (https://www.asahq.org/resources/clinical-information/asa-physical-status-classification-system). Exclusion criteria were hepatic, abdominal, respiratory or cardiac disease, pregnancy or lactation, and treatment with analgesics, respiratory depressants, high sedative doses or sympathomimetic amines within the previous 7 days.

### Premedication and induction of anaesthesia

All animals were premedicated according to the protocol normally used in the participating clinic. This usually incorporated intravenous (i.v.) acepromazine (0.02–0.03 mg/kg bodyweight [bwt]) approximately 1 h before placement of a jugular venous catheter and induction of anaesthesia. Induction was preceded by an alpha_2_ adrenoceptor agonist (alpha_2_ agent) (romifidine 0.08 mg/kg bwt or detomidine 0.01 mg/kg bwt) given i.v. followed immediately by either buprenorphine (Vetergesic Multidose)^1^, (*group BN*, 5–10 μg/kg bwt) or butorphanol (Torbugesic)^2^ (*group BT*, 0.02–0.1 mg/kg bwt) given i.v. according to a randomised treatment chart allocated to each clinic. The assessor (usually the anaesthetist) was blinded and unaware of the identity of the opioid. The test drug was allocated and drawn up by another clinician, the ‘administrator’. Blinding was completed either by adding saline to the syringe of butorphanol immediately prior to injection so that the test drugs’ volumes were the same, or by the administrator giving the injection. Anaesthesia was induced according to the normal clinic protocol, generally with i.v. ketamine (2–3 mg/kg bwt) and diazepam (0.04–0.06 mg/kg bwt). The quality of sedation after the opioid had been given and the quality of induction were scored on a 4‐point simple descriptive scale (SDS) (Table 1).

**Table 1:** Simple descriptive scales for sedation, induction and recovery quality, surgical conditions, ataxia and pain

Variable	Score and description
Sedation	(0) Fully conscious (1) Reduced response to local activity (2) Standing, ataxic, uncaring about stimulation from handling (3) Very ataxic, would fall if moved, oblivious to local surroundings
Induction quality	(0) Ataxic, barely becomes recumbent, danger of injury to horse and handler (1) Goes down but considerable staggering (2) Goes down easily, but some staggering and poor relaxation initially (3) Goes down perfectly
Surgical conditions	(0) Impossible to perform surgery (1) Surgery performed with difficulty (2) Surgery performed adequately, but not fully relaxed (3) Surgery performed easily on well relaxed animal
Recovery quality	(0) Violent and ataxic, numerous attempts to stand (1) Ataxic, numerous attempts to stand (2) Stands up with minimal ataxia, a few attempts to stand (3) Stands perfectly at first attempt
Ataxia	(0) No ataxia. Horse stands and walks normally; is able to turn tightly (1) Mild ataxia. Horse able to walk, but some lack of limb control (2) Moderate ataxia. Horse can walk only with support, staggers but saves itself from falling (3) Marked ataxia. Horse is unable to walk without danger of falling, staggers, falls if turned
Pain	(0) Normal behaviour, eating, alert, interactive (1) A little subdued, eats intermittently, a little apprehensive about interaction (2) Subdued, walks stiffly, occasional snatch at food only, does not interact willingly (3) Very stiff gait, does not eat, glancing at surgical site, may roll, does not interact at all

### Maintenance of anaesthesia

After induction, the trachea was intubated and anaesthesia maintained with a volatile agent, usually isoflurane, vaporised in oxygen. Horses were allowed to breathe spontaneously or ventilated to prevent hypercapnia, following the usual practice of each clinic. Routine monitoring included heart rate (HR), respiratory rate (RR), mucous membrane colour (pink, pale, cyanotic) and invasive arterial blood pressure (ABP), usually measured in the facial artery. End‐tidal anaesthetic agent concentration (ETiso) and carbon dioxide partial pressure (ETCO_2_) (side stream infrared absorption) were also measured in clinics possessing appropriate equipment. Dobutamine was infused i.v. to provide cardiovascular support as indicated by mean APB (MABP). Surgery was carried out according to the clinic's own protocol; the quality of the surgical conditions was scored by the surgeon, unaware of treatment allocation (Table 1). After surgery, horses were allowed to recover under observation, normally in a padded recovery box; sedation and assistance were given, if necessary. Additional drugs including antibiotics, NSAIDs, inotropes, crystalloids, local anaesthetics and additional alpha_2_ agents, benzodiazepines, ketamine and thiopental were given according to practice routine and recorded.

### Recovery and post operative period

The time intervals between premedication and induction of anaesthesia, duration of anaesthesia and time to recovery (from ceasing the volatile anaesthetic administration to first movement, first attaining sternal and first standing) were recorded. The quality of recovery was scored and post operative pain, sedation and ataxia were assessed 1, 2, 3, 4, 5, 6 and 24 h after anaesthesia using a 4‐point SDS (Table 1). Any horse deemed in unacceptable pain (SDS>1) at any time after surgery was given 10 μg/kg bwt buprenorphine i.v. If still in unacceptable pain 30 or more min later, an NSAID or other treatment deemed appropriate by the clinician was given i.v. and recorded. Intestinal activity was assessed post operatively by auscultation of the abdomen and noting faecal production. Any adverse events were documented. A single person performed all assessments for an individual horse.

### Data analysis

The data from horses in the BN group were compared with those in the BT group. The primary outcome measure was post operative pain score, with a null hypothesis that there would be no differences between the groups. Secondary outcome measures were perioperative physiological variables and post operative sedation and ataxia, with a null hypothesis of no differences between groups.

Data from all horses were used in the statistical analysis as virtually all horses received acepromazine, romifidine, ketamine, diazepam and isoflurane. Detomidine substitution for romifidine, midazolam for diazepam and sevoflurane or halothane for isoflurane in a few horses was deemed unlikely materially to affect the results, since these substitutions were all similarly acting drugs from the same drug group and occurred equally in BN and BT. A few missing data points occurred throughout data collection but were both low in number and equally spread between the groups, and therefore were considered unlikely to affect the results.

Single measurements of continuous variables were compared using unpaired *t*‐tests (bwt, age, drug doses and time periods). Non‐normally distributed data were compared using the Mann–Whitney *U*‐test (all SDS), Chi‐squared test (sex, breed and surgical procedure) or Fisher's exact test (dichotomous data). Continuous data collected over time were analysed using repeated measures ANOVA followed by Tukeys’ multiple comparisons test, if appropriate (HR, RR, ABP, ETCO_2_ and ETiso). For statistical purposes, SDS pain scores in animals requiring rescue analgesia were taken as the score awarded at rescue until the end of the assessment period (LOCF). Data are presented as mean ± s.d. unless otherwise stated; P<0.05 was regarded as significant except for multiple comparisons where P<0.01 was used.

One clinic, which anaesthetised almost half of the horses, consistently used the lowest dose of both buprenorphine (5 μg/kg bwt) and butorphanol (0.02 mg/kg bwt) (low dose), whereas the remaining clinics used nearer 10 μg/kg bwt and 0.1 mg/kg bwt, respectively (high dose). Subsidiary statistical analyses were performed to evaluate potential effects of the different doses by comparing these 2 subsets within each group.

Based on previous studies 11, 13, 50 animals per group were required to give 80% power to detect a 25% difference in the proportion of animals in each group with post operative pain scores of 0 or 1. Smaller differences were deemed unlikely to be of clinical significance. Interim power analysis after 74 horses had been recruited indicated that 100 horses was more than necessary and the investigation was completed with a further 15 cases.

## Results

### Horses details, premedication and anaesthesia

Eighty‐nine horses were recruited (Table 2). The vast majority received acepromazine i.v. approximately 1 h before romifidine and the test opioid were given; most also received an NSAID i.v. (Table 2) and antibiotics i.v. or intramuscularly (i.m.). Oral trimethoprim‐sulphonamide was given several hours before anaesthesia in one clinic. There were no differences between the groups in the proportion of animals treated with each antibiotic protocol (P>0.5). All horses were clearly sedated immediately prior to induction after the α_2_ agent and opioid had been given; most horses in both groups scored 2 and there were no sedation scores of 0 (Table 2). Higher opioid doses were associated with deeper sedation and a few horses in each group were noted to be very ataxic with the heavy sedation (Table 2). Anaesthesia was induced approximately 5 min after the opioid injection with diazepam and ketamine. The median induction score in BN was higher than in BT, but the difference did not reach significance owing to a single score 0 induction in the BN group (Table 2). The dose of opioid (low or high) did not affect any premedication/induction results except for sedation score (all P>0.05).

**Table 2:** Horse details, drug administration and perianaesthetic data in 89 horses premedicated with either intravenous buprenorphine or butorphanol before surgery under general anaesthesia

Variable	BN: buprenorphine n = 43	BT: butorphanol n = 46	Between group comparison
Bodyweight (bwt) (mean ± s.d.)	514 ± 116 kg	522 ± 141 kg	*t* test P = 0.8
Age (mean ± s.d.)	8.5 ± 4.8 years	8.0 ± 4.4 years	*t* test P = 0.6
Sex	12 Female (28%) 19 Gelding (44%) 12 Entire male (28%)	12 Female (26%) 25 Gelding (54%) 9 Entire male (20%)	Chi‐square P = 0.6
Breed	14 Thoroughbred 11 Warmblood 5 Sport horse 5 Cob 5 Pony 3 Other	12 Thoroughbred 10 Warmblood 10 Sport horse 5 Cob 3 Pony 6 Other	Chi‐square P = 0.3
Surgery	8 ENT 26 Ortho 4 Castration 5 Superficial	9 ENT 28 Ortho 5 Castration 4 Superficial	Chi‐square P>0.9
Acepromazine	0.03 ± 0.004 mg/kg bwt (n = 42) 1 horse none	0.03 ± 0.006 mg/kg bwt	*t* test P = 0.6
Alpha_2_ agent	Romifidine (n = 37) 0.08 ± 0.01 mg/kg bwt Detomidine (n = 6) 0.01 ± 0 mg/kg bwt	Romifidine (n = 43) 0.08 ± 0.01 mg/kg bwt Detomidine (n = 3) 0.01 ± 0 mg/kg bwt	*t* test P = 0.3
Opioid dose	High 9.7 ± 1.0 μg/kg bwt (n = 24) Low 5.0 ± 0.02 μg/kg bwt (n = 19) Mean 7.6 ± 2.5 μg/kg bwt	High 0.08 ± 0.04 mg/kg bwt (n = 25) Low 0.03 ± 0.02 mg/kg bwt (n = 21) Mean 0.06 ± 0.04 mg/kg bwt	
Diazepam	0.05 ± 0.02 mg/kg bwt (n = 42) 1 horse midazolam	0.05 ± 0.01 mg/kg bwt	*t* test P = 0.3
Ketamine (induction)	2.3 ± 0.2 mg/kg bwt	2.3 ± 0.2 mg/kg bwt	*t* test P = 0.8
Time acepromazine to induction	66 ± 27 min	73 ± 32 min	*t* test P = 0.3
Time opioid to induction	8 ± 5 min (median 5 min)	10 ± 10 min (median 5 min)	*t* test P = 0.2
Anaesthesia	Isoflurane (n = 39) Halothane (n = 3) Sevoflurane (n = 1)	Isoflurane (n = 44) Halothane (n = 2)	Fisher's exact P = 0.4
Ventilation mode	IPPV (n = 36) (84%)	IPPV n = 36 (78%)	Fisher's exact P = 0.6
Dobutamine infusion	33 horses (77%)	35 horses (76%)	Fisher's exact P>0.9
NSAID premedication	Flunixin n = 27 (63%) Phenylbutazone n = 10 (23%)	Flunixin n = 30 (65%) Phenylbutazone n = 10 (22%)	Chi‐square P = 0.7
Supplementary anaesthesia	23 horses (53%)	25 horses (54%)	Chi‐square P>0.9
Alpha_2_ agent for recovery	Romifidine n = 15 (35%) Xylazine n = 9 (21%)	Romifidine n = 14 (30%) Xylazine n = 5 (11%)	Chi‐square P = 0.3
Sedation quality before induction – all horses	Median score 2 Score 2 n = 31 (74%)	Median score 2 Score 2, n = 33 (73%)	MWU P = 0.6
Sedation quality high and low dose effect	High: score 3, n = 8 (33%) Low: score 3, n = 0 (P < 0.001)	High: score 3, n = 10 (42%) Low: score 3, n = 0 (P<0.001)	Chi‐square P = 0.8
Induction quality	Median score 3 Score 3, n = 28 (65%)	Median score 2 Score 3, n = 20 (43%)	MWU P = 0.05
HR: premean ± s.d. (range of mean per horse during anaesthesia)	Pre 36 ± 4 beats/min (33–35 beats/min)	Pre 36 ± 5 beats/min (33–35 beats/min)	2‐way ANOVA P = 0.8
RR: premean ± s.d. (spontaneous: range of mean per horse during anaesthesia)	13 ± 2 per min (5–6 per min) n = 7	12 ± 3 per min (5–6 per min) n = 9	*t* test P = 0.07
ETCO_2_ (ventilated horses) (range of mean per horse during anaesthesia)	(5.7–6.1 kPa) n = 9	(5.5–6.0 kPa) n = 40	2‐way ANOVA P = 0.3
MABP (range of mean per horse during anaesthesia)	25 min: 65 ± 10 mmHg 60 min: 70 ± 12 mmHg (increase P<0.001)	25 min: 67 ± 11 mmHg 60 min: 72 ± 9 mmHg (increase P<0.001)	2‐way ANOVA P = 0.6
ETiso (range of mean per horse during anaesthesia)	n = 26 5 min: 1.1 ± 0.4% 60 min: 1.3 ± 0.3% (increase P<0.001)	n = 29 5 min: 1.1 ± 0.4 60 min: 1.3 ± 0.2% (increase P<0.001)	2‐way ANOVA P = 0.5
Duration of anaesthesia	102 ± 44 min	91 ± 38 min	*t* test P = 0.2
Recovery quality	Median score 2 Score 3 n = 19 (44%) Score 2 n = 17 (40%) Score 1 n = 6 (14%) Score 0 n = 1	Median score 2 Score 3 n = 16 (35%) Score 2 n = 18 (39%) Score 1 n = 11 (24%) Score 0 n = 1	MWU P = 0.4
Recovery time to 1st move	23 ± 11 min	25 ± 12 min	P = 0.4
Recovery time to sternal	29 ± 16 min	29 ± 13 min	P>0.9
Recovery time to stand	36 ± 18 min	35 ± 14 min	P = 0.8
Post operative HR	1 h: 41 ± 12 beats/min 24 h: 39 ± 6 beats/min (decrease P<0.001)	1 h: 38 ± 6 beats/min 24 h: 36 ± 6 beats/min (decrease P<0.001)	2‐way ANOVA P = 0.1
Post operative RR	1 h: 14 ± 5 breaths/min 24 h: 12 ± 4 breaths/min (decrease P < 0.001)	1 h: 14 ± 7 breaths/min 24 h: 12 ± 5 breaths/min (decrease P<0.001)	2‐way ANOVA P = 0.2
Post operative sedation	1 h: median score 1 2 h: median score 0 24 h: median score 0	1 h: median score 1 2 h: median score 0 24 h: median score 0	MWU P = 0.6 (1 h) P>0.9 (24 h)
Post operative ataxia	1 h: median score 1 2 h: median score 0 24 h: median score 0	1 h: median score 1 2 h: median score 0 24 h: median score 0	MWU P = 0.4 (1 h) P>0.9 (24 h)

Breed: Other = Arab, Andalusian, Shire, Quarter Horse.

Surgery: ENT = tieback, tie forward, Hobday, soft palate cautery and eye surgery.

Ortho = orthopaedic procedures including neurectomy, splint bone debridement, tooth extraction and desmotomy. Castration includes cryptorchidectomy.

Superficial = skin mass removal and hernia repair.

Supplementary anaesthesia: 1 or 2 ketamine doses (0.1–0.3 mg/kg bwt), thiopentone (0.5–1 mg/kg bwt), benzodiazepine with ketamine (0.05–0.2 mg/kg bwt) as a single bolus, local anaesthetic block of the surgical site, intra‐articular local anaesthetic or morphine, α_2_ agent or ketamine infusion.

HR, heart rate; RR, respiratory rate; ETCO_2_, end‐tidal carbon dioxide tension; MABP, mean arterial blood pressure; ETiso, end‐tidal isoflurane concentration.

No significant differences between groups.

Pre, before any drugs administered; MWU, Mann–Whitney *U*‐test.

### Maintenance of anaesthesia

Anaesthesia was maintained with isoflurane and mechanical ventilation in the vast majority of horses (Table 2). Respiratory rate decreased below conscious values during anaesthesia in all spontaneously breathing horses. In the ventilated horses, no intergroup differences were detected in selected rate, ETCO_2_ or ETiso (Table 2). Dobutamine was administered in most horses and MABP was maintained above 65 mmHg in all except one, where it decreased briefly to 55 mmHg (Tables 2, 3). Persistent hypertension did not occur; MABP transiently reached 100–105 mmHg in only a few cases. All horses received crystalloid solutions. Mucous membranes were recorded as pink in all horses except for one in BN that was pale (P = 0.5). No cyanotic mucous membranes were noted. Anaesthesia lasted around 1.5 h in both groups (Table 2) and surgical conditions were excellent (score 3) in over 80% of horses in both groups (P = 0.5). The dose of opioid (low or high) did not affect any results relating to maintenance of anaesthesia (all P>0.05).

**Table 3:** Post operative adverse effects in 89 horses premedicated with either intravenous buprenorphine or butorphanol before surgery under general anaesthesia. There was no significant differences between groups

*Box walking after recovery from anaesthesia*				
n	BN: buprenorphine n = 4	n	BT: butorphanol (2 horses)	Fisher's exact P = 0.4240
3	Within 30 min of rescue analgesia with 10 μg/kg buprenorphine Premed was 5 μg/kg bwt. Resolved spontaneously after 2 h. A further 7.5 μg/kg after 12 h had no adverse effect. Premed was 10 μg/kg bwt. Ceased after romifidine 6 μg/kg bwt and butorphanol 8 μg/kg bwt. Premed was 10 μg/kg bwt. Rescue given 4 h after premed. Walking ceased after acepromazine 0.01 mg/kg bwt at 4 h.	1	5 h after premed Premed was 0.02 mg/kg bwt. Resolved spontaneously after 3 h Rescue analgesia with buprenorphine 10 μg/kg bwt no effect on locomotor activity	
1	8 h after premed. Premed 10 μg/kg bwt. Brief period of walking. Resolved spontaneously	1	Started immediately after recovery. Premed was 0.1 mg/kg bwt. Resolved spontaneously in 1 h	
*Post operative colic*				
(1 horse)		(2 horses)		Fisher's exact P = 1
1	Mild impaction morning after surgery. Premed was 10 μg/kg bwt. Resolved after oral fluid and metamizole/butylscopolamine	1	Distended dorsal colon during night after surgery. Premed was 0.1 mg/kg bwt. Resolved after flunixin 1 mg/kg bwt, buprenorphine 10 μg/kg bwt and inhand walking exercise	‐
*Miscellaneous*				
(1 horse)		(1 horse)		
1	Prolonged mild sedation (7 h) after recovery from cryptorchidectomy. Normal thereafter	1	Hypotension during anaesthesia (MABP 55–60 mmHg) unresponsive to dobutamine. Normal recovery	

n = number affected.

### Recovery and post operative period

An elective α_2_ agent was given i.v. to half of the horses in both groups when they were moved into the recovery box (Table 2). Most horses stood in less than 1 h and quality was score 3 in over a third of both groups. There were no significant group differences in quality or duration (Table 2) and results were unaffected by the dose of opioid (low or high) (all P>0.05). Heart and respiratory rates remained within normal limits post operatively and there were no intergroup differences. The dose of opioid (low or high) did not affect either HR or RR post operatively (all P>0.05).

**Figure 1:**
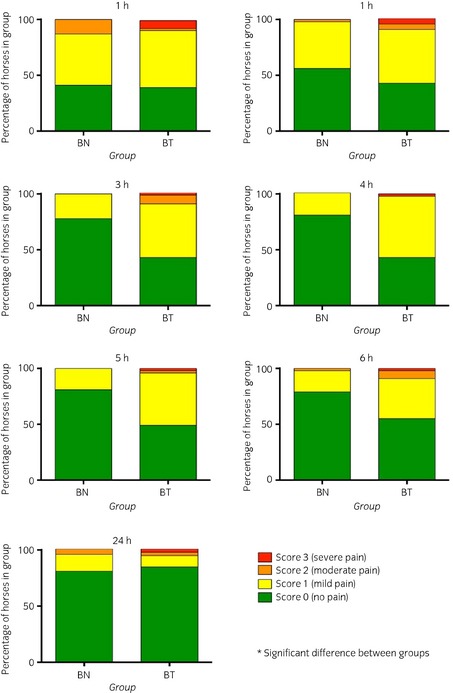
Post operative SDS (0–3) pain scores in 89 horses premedicated with either buprenorphine (n = 43) *group BN* or butorphanol (n = 46) *group BT* before surgery under general anaesthesia. The proportion of the group awarded each score is shown. Significantly more horses received lower scores in BN than BT. *Denotes significant difference between groups:1 h P = 0.9 NSD, 2 h P = 0.2 NSD, 3 h P < 0.001*, 4 h P < 0.001*, 5 h P < 0.001*, 6 h P = 0.01 NSD, 24 h P = 0.5 NSD.

Most horses received a preoperative pain score of 0. A few in each group scored 1 owing to mild lameness of the limb scheduled to undergo surgery (P = 0.6). Post operative pain scores were significantly lower in BN than BT from 3 until 6 h post operatively (Fig 1). Rescue analgesia was given 8.2 ± 6.4 and 8.1 ± 6.3 h (P>0.9), respectively after opioid administration in 5 BN and 11 BT horses (P = 0.2). Of these, 3 BN and 6 BT horses had been premedicated with the low opioid dose (buprenorphine 5 *μ*g/kg bwt; butorphanol 0.02 mg/kg bwt). In all 16 cases rescue analgesia with buprenorphine (7.5–10 μg/kg bwt) was successful, resulting in much more comfortable horses. One horse in BT received phenylbutazone which was also effective. One BN and 5 BT horses required more analgesic treatment and were given a NSAID after a further 12 and 3.2 ± 2.3 h, respectively. This second treatment was effective in all cases. Three horses in BN were given NSAID in the evening after soft tissue surgery, although their pain scores were all 0.

All sedation and ataxia scores were 0 before anaesthesia. Both sedation and ataxia were evident 1 h post anaesthesia in both groups when a few horses in each group were still recumbent. Thereafter, sedation and ataxia abated and the median score for both variables was 0 in both groups (Table 2). A couple of horses in each group were still mildly sedated (score 1) at 4 h and mildly ataxic until 5–6 h but all scored 0 by 6 h except for 2 in BN of which one had been given acepromazine post operatively (Table 3). There was no difference between the groups post operatively in either sedation or ataxia (Table 2).

### Adverse effects

No animals died or suffered any significant perioperative morbidity. Mild box walking and post operative colic occurred in very few horses in either group (Table 3). Box walking could be controlled by normal head restraint and was not extreme.

## Discussion

The investigation fulfilled the aim of comparing the effects of buprenorphine and butorphanol premedication under conventional UK equine anaesthetic clinical practice. The hypothesis that buprenorphine would provide better post operative pain control than butorphanol was supported.

The study was intended to compare the 2 opioids under conditions representing normal UK equine veterinary practice. As a result, a range of anaesthetic protocols, surgical procedures and types of horse were included. The range was equally represented in both groups and the results should be free from bias and applicable to many scenarios encountered in equine practice. In order to retain a normal clinical setting, where a single dose of opioid is commonly used to enhance sedation, no allowance such as repeat dosing was made for butorphanol's shorter duration of action than that of buprenorphine 14. The longer lasting post operative effect of buprenorphine in this study is consistent with experimental data.

The dose range of each opioid was intended to be equivalent and based on the respective datasheet. Buprenorphine recommendations (5–10 μg/kg bwt) were derived from laboratory studies using acute nociceptive threshold testing 14 where comparison was made with a single dose of butorphanol. The datasheet dose range for butorphanol (0.025–0.1 mg/kg bwt) is more historical, but the full range is used clinically.

The drugs used for anaesthesia were consistent throughout the study, with acepromazine, romifidine, ketamine, diazepam and isoflurane being used almost exclusively. This appears to reflect a widespread perception that this is the most common protocol currently used in the UK. Data from the few cases receiving alternatives to the regular drugs were included in the analysis as they were equally distributed between groups and only formed a small minority. Replacement drugs were always of the same type that should have a similar effect. For instance, romifidine was replaced with detomidine; both are α_2_ agents used almost interchangeably in horses. Detomidine is slightly shorter acting and may result in more ataxia in a conscious horse 15 and slightly less hypotension during anaesthesia 16, but in all other respects have similar effects. Diazepam was replaced by midazolam on a few occasions. These are both benzodiazepines of similar duration of effect in horses and have been shown to be indistinguishable when used with ketamine for induction prior to surgery for castration 17. Ketamine was used exclusively for induction, demonstrating its recent almost universal adoption for this purpose in horses. Isoflurane was used almost exclusively for maintenance of anaesthesia. Moreover, all volatile anaesthetic agents cause cardiorespiratory depression in horses, and isoflurane and sevoflurane are similar in this respect; although halothane causes greater reduction in cardiac output, the effect on ABP is similar.

The surgical procedures were elective, covering those routinely undertaken in equine practice. Since emergency surgery was not included, neither major trauma repair nor colic surgery were included. Most procedures covered in this study would probably produce only mild to moderate pain and the hypothesis was not tested under extreme conditions. However, a significant difference between the 2 opioids was demonstrated even when the expected degree of pain was not severe, underlining the belief, supported by the power calculations, that the study was appropriate for comparing the analgesic effect of the 2 drugs. It seems unlikely that butorphanol would be sufficient for severe pain, and buprenorphine has yet to be evaluated fully.

One clinic contributing almost half of the cases used lower doses of both opioids than the other centres. Supplementary analysis of the two subgroups, low (buprenorphine 5 μg/kg bwt, butorphanol 0.03 mg/kg bwt) and high (buprenorphine 9.7 μg/kg bwt, butorphanol 0.08 mg/kg bwt) doses in each of the BN and BT groups again demonstrated better pain control with buprenorphine than butorphanol when the higher doses were used, but there was no difference with the lower doses. These comparisons were underpowered and may have failed to detect a true difference with the lower doses. Comparison of the high and low dose effects within each group did not reveal significant differences, although there was a tendency towards better pain control in BN with the higher dose. This comparison is of questionable validity, as it compared data between different clinics and clinicians as well as being underpowered. However, these results do indicate that even at lower doses buprenorphine is the better analgesic, but appears most effective at doses above 5 μg/kg bwt.

The cardiorespiratory effects of anaesthesia in both groups were unremarkable and reflected the expected course of anaesthesia in horses. Hypotension and respiratory depression are well recognised hazards and the use of dobutamine and mechanical ventilation are well accepted in routine equine anaesthesia 18. The need for dobutamine support was similar in the two groups, suggesting that neither opioid contributed to substantially more cardiovascular depression than is normally encountered during volatile agent anaesthesia in horses. Whether a horse was ventilated or not depended more on the usual clinic protocol than a response to any measure of respiratory depression, and too few horses breathed spontaneously to allow evaluation of any differential effect of the 2 opioids on respiration.

Isoflurane requirements and the need for supplementary anaesthetics were unaffected by the opioid used. The latter contrasts with a recent report of better surgical conditions and less requirement for supplementary anaesthetic ‘top ups’ during castration under i.v. anaesthesia after buprenorphine premedication (5 μg/kg bwt) compared with butorphanol (12.5 μg/kg bwt) 12. Volatile agent anaesthesia and longer duration of surgery appeared to obliterate any potential anaesthetic‐sparing differences between the 2 opioids.

The incidence of adverse effects was low and overall treatment successful in all horses. Subjectively, box walking appeared more persistent with buprenorphine and occurred in 3 horses after rescue analgesia with buprenorphine 10 μg/kg bwt. The data (Table 3) suggest that the buprenorphine dose may be critical; 5 μg/kg bwt appears less likely to cause locomotor activity but needs to be set against the better pain relief from 10 μg/kg bwt. One horse box walked after 10 μg/kg bwt but a subsequent dose of 7.5 μg/kg bwt provided pain relief without locomotion. Evaluation of buprenorphine in horses under research laboratory conditions has always reported some signs of excitement 19, which may be dose related. Sedation was less effective when detomidine (10 μg/kg bwt) was combined with buprenorphine 10 μg/kg bwt rather than with 7.5 or 5 μg/kg bwt; 7.5 μg/kg bwt is perhaps the best compromise 20. Clutton 7 emphasises that the precise dose of opioids is important and that they are less likely to cause side effects when used to treat pain. Other sedative and anaesthetic drugs given during general anaesthesia must also contribute and may have lessened box walking in this series. A few published clinical studies using buprenorphine contribute to this discussion. No locomotor stimulation was reported after buprenorphine (6 μg/kg bwt) was given with a detomidine infusion for laparoscopic surgery in standing horses 21. However, more locomotor activity after castration under i.v. anaesthesia was seen in ponies premedicated with buprenorphine (5 μg/kg bwt) than butorphanol (12.5 μg/kg bwt) 12. Box walking was reported in 4/4 horses after 10 μg/kg bwt buprenorphine were given with a detomidine infusion for standing dental surgery, in contrast to 0/4 which received morphine (0.1 mg/kg bwt) 22. Box walking was not reported in any of 46 horses sedated with detomidine and buprenorphine receiving either 5 or 10 μg/kg bwt 23. The amount of buprenorphine at the effector site presumably relates most closely to the likelihood of any opioid effects. Anaesthesia and surgery themselves may affect how the administered dose reaches and is sustained at the effector site. Both detomidine and butorphanol plasma concentrations were lower in horses when the peripheral α_2_ antagonist MK‐467 was also administered 24. Prevention of detomidine's cardiovascular effects with MK‐467 probably enhances excretion of both detomidine and of other drugs given concurrently, through better hepatic drug delivery. If α_2_ agents lead to higher opioid plasma and effector site concentration, particularly if sustained by infusion rather than a single dose, enhanced and prolonged opioid effects may be inevitable. Further investigation into dose, plasma concentration and effect relationships should characterise appropriate buprenorphine dosage to provide the optimal analgesia without side effects.

Decreased intestinal motility is a well known effect of opioids in horses 25. Previous investigations indicated that preoperative administration of morphine, at least, was not associated with a greater incidence of colic 10. The present study supports this, reporting a similarly low incidence of post operative colic and no association with either μ‐ or κ‐agonist opioid premedication. Numerous other characteristics of anaesthesia and surgery, such as change in management, use of α_2_ agents, perioperative starvation and pain, may predispose to altered intestinal function; all need to be taken into consideration when selecting optimal management for horses undergoing surgery.

In conclusion, buprenorphine (5–10 μg/kg bwt) resulted in better post operative analgesia than butorphanol (0.03–0.1 mg/kg bwt) without causing further physiological disruption than normally expected of general anaesthesia in horses. Side effects were limited after both opioids but efforts to refine the buprenorphine dose would be worthwhile to reduce these further.

## Authors’ declaration of interests

P.M. Taylor is an independent consultant in veterinary anaesthesia who, from time to time, acts as a consultant to veterinary pharmaceutical companies. The study herein was carried out under such an arrangement with Alstoe Animal Health Ltd., UK. The equine clinics where the procedures were carried out were paid a fee per completed record sheet submitted to the study monitor.

## Ethical animal research

The investigation was approved under Animal Test Certificate No. 14094/0006 issued by the Veterinary Medicines Directorate. Informed owner consent w as given in all cases.

## Sources of funding

The investigation was funded by Alstoe Animal Health Ltd. This consisted of supply of the buprenorphine and butorphanal used in the investigation and Dr Taylor's consultancy fees.

## Authorship

P.M. Taylor contributed to the conception, made a substantial contribution to the design, analysis and interpretation of the data, wrote the manuscript and gave its final approval. H.R. Hoare contributed to the design, made a substantial contribution to the acquisition and interpretation of data. A. de Vries made a substantial contribution to the acquisition and interpretation of data, critical revision of the manuscript and its final approval. E.J. Love made a contribution to the design, acquisition and interpretation of data, critical revision of the manuscript and its final approval. K.M. Coumbe contributed to the acquisition and interpretation of the data, critical revision of the manuscript and its final approval. K.L. White contributed to the design and interpretation of the data, critical revision of the manuscript and its final approval. J.C. Murrell contributed to the design and interpretation of the data, critical revision of data analysis and the manuscript and its final approval.

## Manufacturers' addresses

1 Alstoe Animal Health, York, UK.

2 Pfizer Animal Health, Tadworth, UK.
